# Maxillofacial injuries associated with intimate partner violence in women

**DOI:** 10.1186/1471-2458-10-268

**Published:** 2010-05-23

**Authors:** Norkhafizah Saddki, Adlin A Suhaimi, Razak Daud

**Affiliations:** 1 School of Dental Sciences, Universiti Sains Malaysia, 16150 Kubang Kerian, Kelantan, Malaysia; 2 Accident and Emergency Department, Hospital Raja Perempuan Zainab II, 15586 Kota Bharu, Kelantan, Malaysia

## Abstract

**Background:**

The facial region has been the most common site of injury following violent episodes. The purpose of this study was to determine the prevalence and pattern of maxillofacial injuries associated with intimate partner violence (IPV) in women treated at a single facility in Malaysia.

**Methods:**

A retrospective review of 242 hospital records of female IPV victims who were seen at the One-Stop Crisis Centre (OSCC) in Hospital Raja Perempuan Zainab II, Kelantan over a two-year period (January 1, 2005 to December 31, 2006) was performed. A structured form was used for data collection. Information regarding the anatomical sites of injuries, types of injuries, and mechanisms of assault were obtained.

**Results:**

Most victims were married (85.1%), were injured by the husband (83.5%), and had at least one previous IPV episode (85.5%). Injury to the maxillofacial region was the most common (50.4%), followed by injury to the limbs (47.9%). In 122 cases of maxillofacial injuries, the middle of the face was most frequently affected (60.6%), either alone or in combination with the upper or lower third of the face. Injury to soft tissues (contusions, abrasions and lacerations) was the most common (87.7%).

**Conclusions:**

This study indicates there is a high prevalence of maxillofacial injuries associated with IPV among women treated at the OSCC in Kelantan, Malaysia.

## Background

Intimate partner violence (IPV) refers to any behaviour within an intimate relationship that causes harm to one or both of those in the relationship [1]. An intimate partnership is any present or previous intimate relationship, with or without sexual involvement. Thus, IPV can occur between spouses, former spouses, or non-married intimate partners [2]. The term IPV has been used interchangeably with domestic violence (DV) [3]. However, in more recent years, DV has developed a broader meaning, and now includes abuse that occurs in any relationship within a household, including that among children, elders, or siblings [4].

In the past, IPV was considered a personal matter. However, the recognition that IPV occurs in all countries of the world irrespective of social, economic, cultural, or religious values, has made it a worldwide health and human rights issue [5]. The prevalence and severity of IPV against women is substantial. An analysis of 48 population-based studies by the World Health Organization (WHO) indicated that 10% to 69% of women had been physically assaulted by an intimate partner at some point in their lives [1]. Another multi-country study indicated that the lifetime prevalence of IPV was 15% to 71% [6].

IPV has profound consequences on health. Abused women are more likely to have physical and psychological problems, including injuries, chronic pain syndromes, gastrointestinal disorders, reproductive health problems, depression, psychosomatic disorders, and limitations in social functioning [7-9]. In addition to these human costs, IPV is also associated with loss of productivity and increased use of health care and social services, even long after the end of the violent episodes [10,11]. Many cases of IPV are not reported and victims may not respond honestly to sensitive questions by health care providers because of the stigma associated with IPV and because the victim may fear reprisal.

Recognition of acute injury pattern caused by IPV is important to aid in the detection and diagnosis of IPV. Previous studies have shown that areas of the head, neck, and face were the most common sites of injury from IPV episodes with prevalence that ranged from 40% to 81% [3,12,13]. It has also been noted that women who reported to an emergency department with head, neck, and facial injuries were 7.5 times more likely to be victims of violence than women whose injuries were limited to other areas of the body [14]. Thus, an injury to the head, neck, and facial region of women may be an initial marker of IPV.

Comprehensive information on the pattern of injuries associated with IPV is not readily available in Malaysia. The purpose of this study was to determine the anatomical sites and types of injuries, particularly maxillofacial injuries, and mechanisms of assault that are associated with IPV in a sample of women treated at a single facility in Malaysia.

## Methods

### Introduction to the study area

This study was conducted at the One-Stop Crisis Centre (OSCC) of Hospital Raja Perempuan Zainab II (HRPZ II), Kelantan. This facility provides medical, social, and legal assistance to victims of violence. The OSCC was established based on the concept of integrated collaboration and coordinated networking of inter-agency and inter-disciplinary teams for the management of violence. This service is available at all emergency departments of all government hospitals in Malaysia.

### Population and sample

This retrospective study involved examination of the medical records of all IPV cases that were referred to the OSCC from January 1, 2005 to December 31, 2006, either by self-referral or referral from other agencies such as police. IPV was defined as any violence toward a woman by her male intimate partners who is or was in a sexual or non-sexual relationship with the woman. The intimate partner may be a husband, former husband, or non-married partner. The partnership history of the victim and perpetrator was established from a checklist used by the OSCC to record details of victims. This checklist includes questions about background of the victim and the perpetrator, description of the violent act and mechanisms of assault, general physical examination of victim, and plan for management. Records with incomplete entries of partnership history or abuse history were excluded. Over the two-year period, a total of 327 cases were treated at the OSCC, and 242 (74%) were identified as IPV. We excluded 82 cases that involved abuse in other relationships within the family (children, elders and siblings), and three cases that had incomplete background on the perpetrator.

The Research Ethics Committee (Human), Universiti Sains Malaysia approved this study. Permission to review medical records was also provided by the Director of HRPZ II.

### Data collection

A structured form was used for data collection. Information on the profile of the victims and the pattern of injuries (anatomical sites of injuries, types of injuries, and mechanisms of assault) were obtained from the checklist and from medical notes. The anatomical sites of injuries were classified as: head (non-maxillofacial region), maxillofacial region, neck, chest, abdomen, back, buttock, limbs, and other. The types of injuries were classified as: contusion, abrasion, laceration, bite wound, burn, fracture, dislocation, sprain, and other. The mechanisms of assault were classified as: punch, kick, slap, strangle, hit by an inanimate object, knife or sharp penetrating object.

Maxillofacial injuries, defined as injuries to any of the hard or soft tissues of the maxillofacial region, were further classified according to specific facial location, types of injuries, and mechanisms of assault. Data were entered, checked and analysed using SPSS for Windows (version 12.0, SPSS Inc., Chicago) statistical software package. Descriptive statistics (frequencies and percentages) were calculated and reported.

## Results

Table 1 shows the socio-demographic profile of the 242 IPV victims. The age range was 15 to 59 years-old, and most victims were young or middle-aged. Most of the women were married (85.1%) and most of the perpetrators were their husbands (83.5%). A total of 85.5% of the victims had at least one previous episode of IPV. Among the 242 victims, 11.2% were admitted to the hospital and the others were treated as outpatients.

**Table 1:** Socio-demographic profile of IPV victims (n = 242).

Variable	Frequency (%)
Age group (years)	
≤ 20	15 (6.2)
21 - 30	90 (37.2)
31 - 40	87 (36.0)
41 - 50	41 (16.9)
> 50	9 (3.7)
	
Ethnic group	
Malay	226 (93.4)
Chinese	12 (5.0)
Indian	1 (0.4)
Others	3 (1.2)
	
Marital status	
Single	1 (0.4)
Married	206 (85.1)
Divorced	35 (14.5)
	
Perpetrator	
Husband	202 (83.5)
Former husband	39 (16.1)
Boyfriend	1 (0.4)
	
Previous history of IPV	
Yes	207 (85.5)
No	35 (14.5)
	
Injuries requiring admissions	
Yes	27 (11.2)
No	215 (88.8)

Most of the IPV victims sustained injury to the maxillofacial region (50.4%), followed by the limbs (47.9%) (Table 2). Injury to the head was also prevalent (24.4%). Most injuries (89.4%) affected the soft tissues. There were no reported fractures, and only one case of joint dislocation. Other manifestations such as abdominal pain, epistaxis, reduced hearing, and reduced range of limb movement were also substantial (36.4%). The most common method of assault was punching with a fist (56.2%), followed by kicking (38.4%), and slapping (37.2%). Some victims were cut, stabbed, or threatened with a knife or sharp instrument (4.1%), strangled (7.0%), or beaten with blunt objects such as bottle, stick, pipe and helmet (14.0%). More than one method of assault was used in 57.0% of cases.

**Table 2:** Pattern of injuries sustained by IPV victims (n = 242).

Variable	Frequency (%)
Sites of injuries	
Head	59 (24.4)
Maxillofacial	122 (50.4)
Neck	25 (10.3)
Chest	16 (6.6)
Abdomen	8 (3.3)
Back	30 (12.4)
Buttock	14 (5.8)
Limbs	116 (47.9)
Others	2 (0.8)
	
Types of injuries	
Contusion	148 (61.2)
Abrasion	36 (14.9)
Laceration	20 (8.3)
Bite wound	8 (3.3)
Burn	4 (1.7)
Fracture	0 (0.0)
Dislocation	1 (0.4)
Sprain	0 (0.0)
Others	88 (36.4)
	
Mechanisms of assault	
Punch	136 (56.2)
Kick	93 (38.4)
Slap	90 (37.2)
Strangle	17 (7.0)
Hit by an inanimate object	34 (14.0)
Knife/sharp penetrating object	10 (4.1)

Table 3 shows the pattern of injuries in 122 IPV victims who had maxillofacial injuries. The middle third of the face was most commonly involved (60.6%), either alone or in combination with the upper or lower third of the face. Injury to soft tissues (contusions, abrasions, lacerations) was the most prevalent (87.7%). Other injuries (32.8%) include epistaxis and fracture or loosening of teeth. Most of the maxillofacial injuries were caused by punching (59.0%).

**Table 3:** Pattern of maxillofacial injuries sustained by IPV victims (n = 122).

Variable	Frequency (%)
Sites of maxillofacial injuries	
Upper third only	25 (20.5)
Middle third only	42 (34.4)
Lower third only	17 (13.9)
Upper and middle third	12 (9.8)
Middle and lower third	14 (11.5)
Upper and lower	8 (6.6)
All thirds	6 (4.9)
	
Types of injuries	
Contusion	81 (66.4)
Abrasion	16 (13.1)
Laceration	10 (8.2)
Bite wound	0 (0.0)
Burn	0 (0.0)
Fracture	0 (0.0)
Dislocation	0 (0.0)
Sprain	0 (0.0)
Others	40 (32.8)
	
Mechanisms of assault	
Punch	72 (59.0)
Kick	15 (12.3)
Slap	46 (37.7)
Hit by an inanimate object	11 (9.0)
Knife/sharp penetrating object	1 (0.8)

## Discussion

The majority of IPV victims in this study (50.4%) sustained maxillofacial injuries. This is in agreement with previous studies which found that the head, neck, and facial regions were the most common sites of IPV injuries [12,15,16]. A review of data from standardised interviews with 218 women who presented at the emergency department of the San Francisco General Hospital reported that the face was the most frequently assaulted area of the body (68%) [3]. Another study at the emergency department of an inner-city hospital in Portland, Oregon also showed that most IPV victims (81%) sustained maxillofacial injuries [13]. These findings suggest the clinicians to consider the possibility of IPV anytime a woman presents at an emergency department with head, neck, or facial injuries in absence of another known specific cause, such as motor vehicle accident [17].

The face is the most conspicuous and unique part of the body that contributes to an individual's self-image and self-esteem. The reasons an attacker targets the face during IPV assault are largely unknown, although it may be because the face is the most reachable part of the body, or it may be that the attacker consciously or unconsciously seeks to undermine the victim's self-esteem. A cross sectional study of 81 male and 46 female patients who presented at the emergency care centre of the Grady Memorial Hospital in Atlanta, Georgia found that 17 of 75 patients with head, neck, and facial injuries were DV victims and that of 18 DV victims, 17 had head, neck, and facial injuries. The authors concluded that head, neck, and, facial injuries are markers of DV with good sensitivity (95%) but poor specificity (45%) [16]. A subsequent study performed at the same centre on a sample of 100 women showed that 91.2% of DV victims sustained injuries to the head, neck, and facial region, and that 53.5% of women with injuries to the head, neck, and facial regions were victims of DV [14]. Thus, head, neck, and facial injuries appear to be sensitive but non-specific markers of IPV, suggesting that all injured women should be screened for IPV regardless of the sites of injuries [17].

In this study, the middle third of the face was more commonly involved and soft tissue injuries (contusions, abrasions and lacerations) to the maxillofacial regions were highly prevalent. This pattern is similar to that reported in other studies with different populations [13,18]. In this study, the most common mechanism of assault for maxillofacial injuries was being hit or punched by a fist. The common use of fist as a means of assault was reported in other studies [13,15,18]. A previous investigation linked the mechanism of assault with the type of injury, and suggested that attacks with the fist can generate enough forces to cause fractures, particularly if more than three punches were delivered at any one site [18]. Thus, because we observed no fractures in this study, even though the fist was the most common mechanism of assault, it may be that our victims received fewer punches. The high prevalence of soft tissue injuries in this study might be due to the impact of falls following punches or kicks [19]. The exact aetiology of injuries following IPV could not be determined in this retrospective study.

Injury to the maxillofacial region was most common in this study, but there were also a substantial number of limb injuries (47.9%). This is in agreement with other studies of different populations which found that the extremities, particularly the arms, were also common sites of injury [3,13,18]. Presumably, this is due to the natural tendency of victims to defend themselves during the assault.

Abusive behaviours tend to occur repetitively. For the vast majority of the women in this study (85.5%), the reported IPV incident was not the first episode of abuse. Again, this is consistent with previous studies [3,6,12,20]. Only a small number of the victims (11.2%) were hospitalised for treatment of injuries, possibly because most of our victims only sustained soft tissue injuries. However, there were instances in which the victims themselves refused admission, possibly due to fear that their partners would discover them or due to worries about the safety and well being of their children. This underscores that it may be difficult for a victim to leave an abusive relationship [21]. In fact, there are many reasons why a woman might stay in an abusive relationship, including fear for safety, concern for her children, lack of alternative financial support, emotional dependence, unwavering hope that the partner will change, and lack of support from family and friends [1]. Thus the role of health care providers in the identification, assessment and response to IPV is pivotal, not only because of the impact of violence on health, but because health care providers may be the only ones who provide help and support for victims [22].

The WHO considers the response of health care services to victims of violence an international priority [5]. However, health care providers are not typically qualified to advise victims of IPV about their relationships. In fact, ill-informed advice may put the women in greater danger. It has been shown that women who leave their partners may face an increased risk of assault [23]. The role of health care providers is essentially to identify IPV and provide information about where victims can go for help [24]. However, previous studies have shown that health care providers generally do not have the knowledge necessary for identification and management of IPV [25-28]. In particular, they are not confident to ask sensitive questions, are not aware of available agencies that support IPV victims, have problems with personal perceptions and feelings about IPV, and are constrained by institutional barriers, such as the lack of protocol in IPV management and the lack of time, space, and privacy. Informational and personal affective barriers have been considered the most significant of these factors [29]. Education and training of health care providers and development of policy statements and standard protocols regarding IPV were thus recommended [30]. The high prevalence of facial injuries following IPV indicates the unique role of oral health professionals in the detection of IPV and management of victims. However many oral health professionals are unaware of this association and their interventions have been reported to be minimal [31,32].

This study highlights important features of the pattern of injuries associated with IPV. In addition, we note with some distress that the number of IPV cases treated in the HRPZ II OSCC during the two year period of our study far exceeded the total number of DV cases (168 DV cases, including that of other relationships within a household) reported by the police for the whole state of Kelantan over the same time period. The OSCC may report more cases of IPV than the police because victims are more familiar and comfortable with health services and feel more open towards health care personnel, whereas going to the police may create discomfort or stigmatisation. In Malaysia, the national crime and violence statistics are mainly provided by the Royal Malaysian Police Department and the report is considered the most comprehensive compared to that collected by other agencies such as the Social Welfare Department [33]. However, in spite of a well-coordinated data collection system, many crimes, particularly incidents of IPV, are not reported. A national survey conducted by the Women's Aid Organisation (WAO), a non-governmental organisation actively involved in DV prevention activities in Malaysia, estimated that about 1.8 million women over the age 15 were beaten by their husbands or boyfriends in the year 1989. However, only 909 police reports of DV were filed in that year, suggesting an underreporting rate of 99.95% [34]. The results of our study highlight the vital role of OSCC in providing reliable statistics on IPV. We suggest that the Ministry of Health Malaysia consider taking a lead role in developing an integrated data collection system that provides more accurate and comprehensive data on IPV in this country.

The availability of a standard OSCC checklist to record abuse episodes greatly facilitated data collection in this study. This minimised the problem of missing data, a common problem in retrospective studies. On the other hand, the use of secondary data imposed a limitation, in that, the reliability, accuracy and integrity of the records were uncertain. In addition, it should be noted that victims who came forward were mainly women who sustained physical injuries. Social stigma or reluctance to reveal information related to sexual or emotional abuse may have prevented more women from seeking assistance from the OSCC.

## Conclusions

This study found that maxillofacial injury was the most common type of injury associated with IPV in a Malaysian medical centre. We suggest that oral health professionals assume more responsibility in responding to IPV and take more active roles in the detection and management of IPV. Training of oral health professionals and other health care providers appears to be necessary to underline their professional roles and to guide them in providing services to victims of IPV.

## Competing interests

The authors declare that they have no competing interests.

## Authors' contributions

NKS contributed to the design of the study, data analyses and interpretation, and wrote the manuscript draft. AAS and RD contributed to the data acquisition and analyses and interpretation of results. All authors participated in revision of the manuscript. All authors read and approved the final manuscript.

## Pre-publication history

The pre-publication history for this paper can be accessed here:

http://www.biomedcentral.com/1471-2458/10/268/prepub
